# Use of UKCAT scores in student selection by UK medical schools, 2006-2010

**DOI:** 10.1186/1472-6920-11-98

**Published:** 2011-11-24

**Authors:** Jane Adam, Jon Dowell, Rachel Greatrix

**Affiliations:** 1 Hull York Medical School, University of York, York YO10 5DD, UK; 2 Tayside Centre for General Practice, Mackenzie Building, Dundee Medical School, Dundee, DD2 4BF, UK; 3 UKCAT, Faculty of Medicine and Health Sciences, Queen's Medical Centre, Nottingham NG7 2UH, UK

## Abstract

**Background:**

The United Kingdom Clinical Aptitude Test (UKCAT) is a set of cognitive tests introduced in 2006, taken annually before application to medical school. The UKCAT is a test of aptitude and not acquired knowledge and as such the results give medical schools a standardised and objective tool that all schools could use to assist their decision making in selection, and so provide a fairer means of choosing future medical students.

Selection of students for UK medical schools is usually in three stages: assessment of academic qualifications, assessment of further qualities from the application form submitted via UCAS (Universities and Colleges Admissions Service) leading to invitation to interview, and then selection for offer of a place. Medical schools were informed of the psychometric qualities of the UKCAT subtests and given some guidance regarding the interpretation of results. Each school then decided how to use the results within its own selection system.

**Methods:**

Annual retrospective key informant telephone interviews were conducted with every UKCAT Consortium medical school, using a pre-circulated structured questionnaire. The key points of the interview were transcribed, 'member checked' and a content analysis was undertaken.

**Results:**

Four equally popular ways of using the test results have emerged, described as Borderline, Factor, Threshold and Rescue methods. Many schools use more than one method, at different stages in their selection process. Schools have used the scores in ways that have sought to improve the fairness of selection and support widening participation. Initially great care was taken not to exclude any applicant on the basis of low UKCAT scores alone but it has been used more as confidence has grown.

**Conclusions:**

There is considerable variation in how medical schools use UKCAT, so it is important that they clearly inform applicants how the test will be used so they can make best use of their limited number of applications.

## Background

For many years there have been three stages in the selection of students for UK medical schools: assessment of academic qualifications and further qualities obtained from the UCAS (Universities and Colleges Admissions Service) application form, usually leading to invitation to interview, and selection for offer (either after or without interview). Medical schools must use legitimate criteria to discriminate between applicants and make an unequivocal offer/reject decision about each applicant. Schools use different, locally-devised methods of assessing and ranking applicants in order to make this decision.

In 2005, 23 medical schools and 8 dental schools began collaboration in the development of United Kingdom Clinical Aptitude Test (UKCAT) [1]. Three more medical schools and one more dental school joined the Consortium in 2007. The test, as introduced in 2006, consisted of four cognitive subtests (measuring verbal reasoning, quantitative reasoning, abstract reasoning and decision analysis), providing four subtest scores and an overall total score for each candidate. An additional non-cognitive test was added in 2007, but the results were not provided to the schools for use in selection and will not be considered further here. Each cognitive subtest was marked based on the number of correct responses a candidate made. There was no negative marking for incorrect answers. The number of correct responses was transformed into a scaled score and presented to candidates once they had completed the test. This final scaled score for each subtest has a range from 300 to 900, with a mean score of around 600. If added together, the four subtest scores give an overall UKCAT score between 1200 and 3600.

The UKCAT cognitive test scores provided a standardised and objective tool that all schools could use to assist decision making, either at the invitation to interview stage, or at the offer stage, or both. The UKCAT Consortium agreement enabled participating schools to decide how best to use the UKCAT within their own selection process, having been informed about the content, scoring and statistical performance of the test [2].

This paper describes the patterns and changes in the use of the UKCAT cognitive scores by UK medical schools in student selection over the four years of testing, 2006 to 2009 inclusive (i.e. for students admitted to medical school 2007-2010 inclusive), based on an annual telephone survey of admissions conveners of all participating schools. The purpose is to inform debate regarding UKCAT and medical admissions in general as well as the design of studies on the impact and validity of this new test. Dental schools' data will be reported separately.

## Methods

Annual retrospective key informant telephone interviews were conducted with every UKCAT Consortium medical school, using a pre-circulated questionnaire (Table 1). Interviews were conducted in the summer terms of each admissions cycle. The interviewers (JA in 2007-9 and RG in 2010) were familiar with the medical admissions and selection systems. The main focus was on selection for each school's standard five-year undergraduate course, but the study also includes three schools that only used UKCAT to select for their graduate-entry course. The respondent, a senior member of the admissions staff at each school, provided a detailed, confidential description of their school's selection processes the year before the introduction of the UKCAT, and every subsequent year, to allow an accurate evaluation of the use and impact of the UKCAT. The schools also commented on whether any major change was seen in the population to whom offers were made, and whether the UKCAT was more useful when assessing any particular subgroups of applicants. The key points of the interview were transcribed, a summary was sent to each informant and then an agreed corrected version was returned for analysis ('member checking'). A content analysis was undertaken by JA and RG, and repeated independently by JD, leading to an agreed categorisation of the range and frequency of ways that the UKCAT results were used [3], in the context of the different criteria and processes by which the medical schools selected their students [4].

**Table 1:** Telephone Questionnaire

1		Please describe your selection process before you introduced the UKCAT (year 1)/this year (year 2 onwards).
**2**		How did you use the UKCAT result in your selection process this year?
	
	**2a**	Confirm if used for • Pre-selection • Selection for interview • After interview
	
	**2b**	Did you use it for assessment or selection of any specific subgroups (for example borderline, WP, mature or disabled)? Please specify and describe.

**3**		Did you make any other changes to your selection process this year?

**4**		Do you think using the UKCAT has affected the profile of the candidates you have selected this year, compared with previous years?
	
	**4a**	If so how?
	
	**4b**	Do you have any evidence to support this?

**5**		Have you analysed (or do you intend to analyse) the effect of using the UKCAT on the profile of your selected candidates this year?
	
	**5a**	If so, how?

**6**		Will you change the way in which you use the UKCAT in your selection process next year?
	
	**6a**	Will you change other aspects of your selection process next year as a result of using the UKCAT?

Name of informant Role of informant

Tel no E mail Interviewer

Date of interview Start time End time/duration

Individual schools used different forms of the UKCAT score, such as the total test score, the individual subtest scores, the average subtest score, and transformations of the score such as the applicant's percentile, decile or quartile ranking in the total population of UKCAT candidates for that year (or in the applicant pool to that particular medical school). For simplicity these will all be referred to as the 'UKCAT score' throughout; to allow comparisons between schools all scores have been converted to the equivalent of the total UKCAT score, which is the sum of the four subtest scores.

## Results

Although each school used the UKCAT score slightly differently, four main methods could be distinguished, shown as borderline, factor, threshold and rescue in table 2. The characteristics of each method are described below. Preferred methods emerged over the years, and almost a third of schools used more than one method, at different stages in their selection cycle.

**Table 2:** Summary of medical schools' uses of UKCAT scores

Year	Total n of schools	Method				More than one method
		**Borderline**	**Factor**	**Threshold**	**Rescue**	

**2006**	23	13	7	1	7	7

**2007**	26	8	10	9	7	8

**2008**	26	9	11	10	8	10

**2009**	26	10	12	10	7	10

### Borderline method

The 'borderline' method was employed when schools used the UKCAT score to discriminate amongst a small number of applicants lying at a decision borderline, who were otherwise indistinguishable (on the school's other selection criteria) and were too numerous to be treated the same (i.e. made an offer or rejected). Candidates' UKCAT scores were then used as the only objective basis for discrimination within the group, with higher scoring applicants being made an offer (of an interview or a place) and the lower scoring ones rejected. Characteristically, therefore, using the borderline method meant that the UKCAT scores only contributed to the selection decision for a small proportion of applicants, estimated by different schools to range from just a few to no more than 100 candidates (mean 30, median 5).

In 2009, ten medical schools used the Borderline method at the offer stage. Four of the thirteen schools that initially used the borderline method had switched to using a threshold score, three were using a factor method and four had added in a rescue trade-off. Only two schools continued to use the borderline method alone.

### Factor method

Twelve schools used the factor method in 2009. In most cases schools factored in (or added) every applicant's UKCAT score (or a proxy for that score) to the score the applicant obtained from the school's usual method of assessment, to provide a total score. The total score then determined the outcome/reject decision for each applicant (for an interview or a place, depending on the stage in the selection cycle). In some cases a more sophisticated matrix of factors was utilised or schools combined rankings of different factors including the UKCAT. The important characteristic of this method is that all applicants UKCAT scores were used, across the whole range of UKCAT scores, but only as one of a range of factors contributing to the final decision.

In 2009 the weight given to the UKCAT score in medical schools' overall selection processes ranged from 2% to 33%. Schools could use the UKCAT score either at the stage of making the decision to interview (mean 17%) or for making the decision to offer a place (mean 9%). In 2009 four schools used it only for invitation to interview, four schools used it only to make offers, and four schools in effect used it at both stages, because the applicant's pre-interview assessment score (which included the factor from the UKCAT score) was carried over and added to the applicant's interview score, to reach a final score that was used to make the decision to offer a place.

Most schools using a factor method have used the same weightings since introducing the method. The weighting of the UKCAT score used at the selection for interview stage tended to be higher than that used at the offer stage. This was because at the offer stage the additional information about the candidate's performance at interview was also factored in.

### Threshold method

A minimum or threshold UKCAT score was adopted by some schools to create a hurdle that the applicant must cross to reach the next stage in the selection process, usually selection for interview. The hurdle was introduced either after assessment of the academic qualifications or after assessment of the UCAS form. The height of the hurdle (the required minimum score) was either predetermined, or determined by convenience depending on the number of applications received, so set number of applicants proceeded to the next stage. Some schools used total score and some used subtest scores, and in 2007 only three subtest scores were available. Comparisons between schools and years have therefore been made after standardising all threshold scores to the total score derived from all four subtests. Whereas only one school applied a threshold in 2006, this approach had been adopted by 10 schools by 2009. Four of the ten schools made a detailed assessment of the UCAS forms before applying the UKCAT threshold to the better applicants only. Six schools applied the threshold to all applicants meeting the school's academic criteria and immediately invited the applicants above the threshold to interview. In the latter group the UCAS form was utilized later in the selection process.

Threshold scores have ranged over time from 1800 to 2730. Over the years the mean average threshold score used has risen from 2350 (median 2350) in 2006 to 2521 (median 2600) in 2009.

### Rescue method

Some schools first scored each application using their own standard method of assessment but then allowed a UKCAT score above a pre-determined level to 'trade-off' against or compensate (normally by a fixed amount) for a lower score in some part of that assessment. This then led to a positive decision to offer (either an interview or place) rather than to reject that applicant. In some cases, schools automatically invited to interview candidates with scores > n or candidates above a certain percentile.

The trade-off approach therefore 'rescued' applicants who would otherwise (or previously) have been rejected. Determining the number of applicants affected by these methods has not always been possible especially where a trade-off matrix was utilised. However where data was available the estimated number of applicants rescued by this strategy in different schools ranged from one to 100 (mean 35, median 40). The key characteristic of the trade-off method that distinguished it from the factor method was that the school pre-determined the scores at which the trade-off would be applied and the amount of compensation to be given. Seven schools used this method in 2009. The number of candidates 'rescued' through this method has remained fairly constant over the four years of the test.

### Multiple use of UKCAT scores

Some schools used the UKCAT scores in more than one way at different stages in the selection process, to help select either for interview, or for offer of a place (either a formal offer or offer of a place on the school's informal waiting list), or both. For example in 2009 two schools that used the factor method also used the UKCAT score to provide a rescue trade-off at another point in the selection cycle, while six schools that used the rescue method also used the borderline method at a later stage. The frequency of use of each method at the different stages of selection is shown in table 3. This includes data from schools that used the same method at more than one point in the selection cycle, and those that did not routinely interview.

**Table 3:** Method of use of UKCAT score at different selection stages

	Borderline		Factor		Threshold		Rescue	
**Year**	**Interview**	**Offer**	**Interview**	**Offer**	**Interview**	**Offer**	**Interview**	**Offer**

**2006**	1	13	4	4	1	0	7	0

**2007**	0	8	6	7	8	1	7	0

**2008**	0	9	7	7	9	1	8	1

**2009**	0	10	8	7	10	0	7	0

A small number of schools have used the test in three ways during the admissions' process. For example one school used it as a factor method to select for interview, then as a rescue method for candidates who had scored highly but not made the threshold for interview invite. They then used it a third time as a borderline method to identify candidates for offer following interview.

## Discussion

The main aims of introducing the UKCAT were to provide schools with a tool that would offer a more objective and fairer method of discriminating between academically high achieving applicants, and support widening participation initiatives. A subsidiary, but very important aim, was to establish the predictive validity of these tests in identifying successful doctors. In the first year the schools were unfamiliar with the test and its psychometric characteristics and were concerned not to use this new tool heavy-handedly. Many schools therefore chose the borderline method because it would affect the outcome for only a small number of applicants where there was no other logical approach to use. The factor method was also seen as a fair but 'light-touch' means of using the UKCAT score as part of the selection decision.

The threshold method, like the factor method, ensured every applicant's UKCAT score contributed to the offer/reject decision. The threshold method was used in 2006 by only one school, where it was intended as a 'widening participation' tool, because the school at the same time reduced the level of academic pre-requisites. Most schools using this method by 2009 were attracted by the opportunity for speedier and more efficient selection of applicants for interview, by postponing detailed consideration of applicants' UCAS form until a later stage. This may be justifiable in the light of studies [5,6] suggesting that the UCAS personal statement is a poor predictor of the subsequent medical performance of students once they have been selected. However, reducing the importance of the UCAS form represents a shift from the popularly understood use of this information in selection for medical school.

The rescue trade-off method was used to increase the pool of applicants invited to interview, for example by including those with high UKCAT scores (indicating high ability) but weaker UCAS applications (perhaps because of lack of advice in preparing their personal statement). This method thus appears the most explicit 'widening participation' use of the UKCAT score. In keeping with the schools' desire not to penalise applicants on the basis of their UKCAT score, it is noteworthy that no school employed the alternative strategy of a 'reject' trade-off, whereby a low UKCAT score would lead to the rejection of an applicant who would formerly have been selected on the basis of the school's usual assessment score.

Many schools had decided to keep their use of the UKCAT scores constant for the first few years and then review their experience. As familiarity grew with the UKCAT, schools increased the number and combinations of ways in which they used the test, or the weight the results were given. Some schools reported local analyses had indicated UKCAT scores mirrored the outcomes of their existing selection methods and consequently their confidence in the use of the test. At the same time the Consortium acted as a forum for sharing innovation and best practice, helping members to develop and change their use of the test.

Many schools reported that the UKCAT was particularly helpful as part of the assessment of non-traditional applicants such as overseas students or mature students, and in the assessment of applicants for medical courses other than the standard five year course, such as Foundation or six year programmes. Where candidates in these groups might be offering a variety of non-traditional qualifications, the UKCAT provides a standardized assessment for comparison.

An understanding of how schools have used the test and how this use has changed since UKCAT's inception is important for those researching the impact of the test on the demographics of medical admissions and the predictive validity of the test. The impact of the use of the test on widening participation in medical admissions is currently being investigated. The use of different methods may well restrict or widen the range of UKCAT scores available which is important for subsequent research into predictive validity.

The findings of this study, with 100% completion in each year, are important for informing debate on medical admissions, especially in the UK. They also highlight the considerable variation in practice which has now emerged and, therefore, the importance of transparency on the part of schools and informed decision making by applicants once they know their UKCAT score. UKCAT will remain contentious unless or until convincing evidence emerges regarding predictive validity but already some medical schools are choosing to place more reliance on the UKCAT score than on the UCAS form statements. This is because there is insufficient evidence of the predictive power of the UCAS statement [7], because assessors have concerns that unequal levels of support provided to applicants in writing the statement, and because of the difficulties of detecting plagiarism and deception. The UKCAT Consortium has commissioned an initial study into the predictive validity of the cognitive sections of the test,

## Conclusions

This study reports the use of UKCAT scores in selection of applicants for the main medical programme of each of the 26 medical schools in the UKCAT consortium. The methods depended respectively on using the score to make only **borderline **decisions, using the score as one **factor **added to a range of other factors, setting a **threshold **UKCAT score in order for the application to progress, and using a high UKCAT score to **rescue **an otherwise weaker applicant.

By 2009 all four methods were used with similar frequency, by nine or ten of the 26 UK medical schools in the UKCAT consortium. Many schools used more than one method, at different stages in the selection cycle. It is therefore important that each medical school sets out clearly how it intends to use the test, and that applicants seek this information beforehand in order to make best use of their limited number of applications.

## Competing interests

JA was an unpaid member of the Executive Board of the UKCAT Consortium from 2006 to 2010, and JD is a current unpaid member. Rachel Greatrix is the (paid) Chief Operating Officer of the UKCAT Consortium. JA received a fee of £5000 from the UKCAT Consortium for undertaking this study.

## Authors' contributions

JA and JD designed the study. JA conducted the interviews (2007-9), undertook the primary analysis of the data and wrote the first draft of the paper. JD independently reviewed the data and commented on the paper. RG conducted the interviews in 2010, undertook the primary analysis of this data and produced further drafts of the paper. All three authors approved the final manuscript.

## Authors' information

JA was Associate Dean for Admissions at Hull York Medical School 2003-2011.

JD is Admissions Convenor at Dundee Medical School.

RG is the Chief Operating Officer of the UKCAT Consortium.

## Pre-publication history

The pre-publication history for this paper can be accessed here:

http://www.biomedcentral.com/1472-6920/11/98/prepub

## References

[B1] UKCAT Board UKCAT Annual Report 2006 http://www.ukcat.ac.uk/pdf/AnnReport2006Web.pdf

[B2] Miller T Guidelines for Using UKCAT Test Scores 2008 London: Pearson VUE

[B3] Patton MQ Qualitative Evaluation and Research Methods 2002 3 London: Sage

[B4] Parry J Mathers J Stevens A Admissions processes for five year medical courses at English schools: review BMJ 2006 332 1005 1008 10.1136/bmj.38768.590174.55 16543300 PMC1450044

[B5] Ferguson E James D O'Hehir F Sanders A Pilot study of the roles of personality, references and personal statements in relation to performance over the five years of a medical degree BMJ 2003 326 429 432 10.1136/bmj.326.7386.429 12595384 12595384 PMC163931

[B6] McManus IC Powis DA Wakeford R Intellectual aptitude tests and A levels for selecting UK school leaver entrants for medical school BMJ 2005 331 555 559 10.1136/bmj.331.7516.555 16150766 PMC1200591

[B7] Wright SR Bradley PM Has the UK Clinical Aptitude Test improved medical student selection? Medical Education 44 1069 1076 10.1111/j.1365-2923.2010.03792.x 20946477

